# Decision-making deficits in obsessive-compulsive disorder are associated with abnormality of recency and response consistency parameter in prospect valence learning model

**DOI:** 10.3389/fpsyt.2023.1227057

**Published:** 2023-09-29

**Authors:** Keitaro Murayama, Hirofumi Tomiyama, Aikana Ohno, Kenta Kato, Akira Matsuo, Suguru Hasuzawa, Kenta Sashikata, Mingi Kang, Tomohiro Nakao

**Affiliations:** ^1^Department of Neuropsychiatry, Kyushu University Hospital, Fukuoka, Japan; ^2^Department of Neuropsychiatry, Graduate School of Medical Sciences, Kyushu University, Fukuoka, Japan; ^3^Integrated Center for Educational Research and Development, Faculty of Education, Saga University, Saga, Japan; ^4^Graduate School of Human-Environment Studies, Kyushu University, Fukuoka, Japan; ^5^Center for Health Sciences and Counseling, Kyushu University, Fukuoka, Japan

**Keywords:** obsessive-compulsive disorder, decision-making, Iowa gambling test, prospective valence learning model, consistency parameter

## Abstract

**Background:**

Patients with obsessive-compulsive disorder (OCD) have deficits in decision-making in the Iowa Gambling Task (IGT). However, no study has investigated the parameters of the prospect valence learning (PVL) model in the IGT for OCD.

**Aims:**

This study aimed to investigate deficits in decision-making in OCD using the PVL model and identify whether the parameters of the PVL model were associated with obsessive-compulsive severity.

**Methods:**

Forty-seven medication-free patients with OCD were compared with 47 healthy controls (HCs). Decision-making was measured using the total net and block net scores of the IGT. A PVL model with a decay-reinforcement learning rule (PVL-DecayRI) was used to investigate the parameters of the model. Correlation analysis was conducted between each parameter of the PVL-DecayRL and obsessive-compulsive symptoms.

**Results:**

The total net score of patients with OCD was significantly lower than that of the HCs. The block net scores of the OCD group did not differ across the five blocks, whereas in the HCs, the fifth block net score was significantly higher than the block net scores of the first and second blocks. The values of the recency and response consistency parameters of the PVL-DecayRI in patients with OCD were significantly lower than those in HCs. The recency parameter positively correlated with the Y-BOCS obsessive score. Meanwhile, there was no correlation between consistency parameter values and symptom severity in OCD.

**Conclusion:**

Our detailed analysis of the decision-making deficit in OCD suggests that the most recent outcome has a small influence on the expectancy of prospect valence, as indicated by the lower recency parameter, and is characterized by more impulsive choices, as indicated by the lower consistency parameter.

## 1. Introduction

Obsessive-compulsive disorder (OCD), according to the Diagnostic and Statistical Manual of Mental Disorders, Fifth Edition (DSM-5), is “*characterized by recurrent, intrusive, and distressing thoughts (obsession) and repetitive behaviors or mental acts (compulsions) that are executed to avoid anxiety or neutralize obsessions”* (1). Several studies have shown that OCD is associated with cognitive impairment (2, 3). Some domains of cognitive impairment, decision-making, inhibition, and planning have been reported as candidates for the endophenotype of OCD (4, 5). A deficit in decision-making refers to the difficulty in selecting an option from various choices that yield different consequences (6). Obsessive-compulsive symptoms include deficits in decision-making because seeking immediate relief from negative emotions such as anxiety and disgust through compulsive behavior results in long-term functional problems (7–9).

There are two types of decision-making in some situations (10). The first includes decisions under risk, and the second includes decisions under ambiguity (10). The former is a decision situation in which information on the results of diverse choices and their prospects is provided. The latter, which is usually measured by the Iowa Gambling Task (IGT) (11), requires decision-makers to find information by themselves by processing feedback of the previous choice in a situation in which the outcome and probabilities are implied but not directly expressed.

The IGT is a task in which participants select cards to win prizes. It required the participants to select one deck from four decks (A, B, C, and D). Decks A and B are disadvantageous decks that cause greater losses than gains. The other decks (C and D) are advantageous, making more gains than losses. Although normal decision-makers select advantageous decks more often than disadvantageous decks as trials progress, patients with OCD do not show this phenomenon (12–16). Therefore, patients with OCD are considered to have deficits in decision-making (4, 15–18).

IGT consists of complex interactions among motivational, cognitive, and response processes (19). Therefore, deficits in decision-making in specific participant groups may result in different component processes in the IGT (19). Some cognitive models have examined the mechanisms of the psychological processes involved in IGT. Two of these, the expectancy-valence learning model (EVL) (20) and the prospect valence learning model (PVL) (21), were successfully fitted with empirical data (19). The EVL model of IGT performance assumes three processes, namely, motivation, memory/learning, and response consistency (22). The motivational parameter shows how the participant integrates the gains and losses of all previous trials and forms an expected valence for the present trial, which depends on the probability of a subsequent trial (22, 23). The memory/learning parameter indicates that the expectancy of a deck in a given trial is the sum of the expectancy of the deck in a previous trial and the difference between the valence of the present choice and that of the deck in a previous trial (22, 23). Response consistency shows the degree of consistency in making decisions based on the expectation of valence for each deck or whether the participants chose decks randomly (22, 23).

The PVL model, which is a modified EVL model proposed by Ahn et al. (21), has four parameters, namely, feedback sensitivity, loss aversion, recency, and response consistency. The motivation component of the EVL model is separated into two parameters as follows: feedback sensitivity and loss aversion (22). Since the PVL model employs a non-linear utility function and effectively explains the impact of gain–loss frequency on the formation of expectancy for each deck, it would be more effective than the EVL model, which uses a linear function for analyzing IGT performance (21). Feedback sensitivity, indicated by the non-linear model, shows the association between the total of gains and losses and prospect valence. A higher feedback sensitivity score indicates that the subjective estimation is more closely related to the actual amount of gains and losses. The loss aversion parameter indicates the inclination to avoid loss. The recency parameter refers to the shaping or adjustment of a preference for each deck, formed by recent outcomes with gains or losses. A low recency parameter indicates that the value of the most recent card choice has a small effect on the expectancy of the deck, and forgetting is more progressive. The response consistency parameter indicates the consistency of the participants' choice behavior. A low response consistency parameter indicates that the participant chose more randomly.

Several clinical studies have used the PVL model to analyze IGT performance. For example, chronic cannabis use was significantly lower in four parameters, especially in loss aversion and consistency, compared with healthy controls (24). All four parameters were significantly lower in patients with schizophrenia than in healthy controls (25). Patients with anorexia nervosa have a significantly lower recency parameter (22), and female students at high risk for anorexia nervosa have a lower recency parameter and response consistency than healthy controls (23). In patients with a major depressive episode with a suicide attempt, the loss aversion and recency parameters were lower than those in controls (26).

Mathematical models allow the decomposition of IGT performance into distinct components: feedback sensitivity, loss aversion, learning (recency), and response processes. This approach enables a comprehensive analysis of the underlying decision-making processes and characterization of decision-making deficits in individuals with OCD. However, to the best of our knowledge, no study has explored these aspects of the PVL model using IGT among individuals with OCD in comparison with healthy controls (HCs).

This study aimed to explore which parameters of the PVL model in IGT showed differences between patients with OCD and healthy controls and identify whether the parameters of the PVL model were associated with obsessive-compulsive severity. Our hypothesis postulated that IGT performance among individuals with OCD would be poorer than HCs. This can be attributed to reduced feedback sensitivity, recency, and consistency parameters among individuals with OCD. Moreover, we hypothesized that the loss aversion parameter in individuals with OCD would be significantly higher compared with HCs. Several previous studies have indicated that individuals with OCD demonstrate higher avoidance learning and loss aversion (27–30).

## 2. Materials and methods

### 2.1. Participants

Forty-seven medication-free patients with OCD (OCD) and 47 HC were recruited. All individuals diagnosed with OCD were recruited from the outpatient unit in the Department of Neuropsychiatry, Kyushu University Hospital, from February 2016 to September 2021. All patients with OCD were diagnosed using the Structured Clinical Interview for Diagnostic and Statistical Manual of Mental Disorders, Fourth Edition (DSM-IV), Axis I disorders (patient version) and fulfilled the DSM-IV criteria. None of the patients had any current comorbid Axis I disorders. We also verified that all patients met the criteria for OCD in the Diagnostic and Statistical Manual of Mental Disorders, Fifth Edition (DSM-5). All OCD participants had not taken any psychiatric medication for at least 4 weeks. These procedures were performed on the same day as the IGT assessment. The HCs were recruited from local communities. They were assessed using the Structured Clinical Interview for DSM-IV (non-patient version). The participants had no history of psychiatric disorders, head injury, or epilepsy.

This study was conducted according to the principles of the Declaration of Helsinki and was approved by the Kyushu University Ethics Committee. All participants provided written informed consent prior to the commencement of the study.

### 2.2. Clinical assessment

The severity of OCD symptoms was measured by the Japanese version of the Yale–Brown Obsessive-Compulsive Scale (Y-BOCS). The Hamilton Rating Scales of Anxiety (HAM-A) (31) and Depression (HAM-D) (32) were used to assess anxiety and depression, respectively. The Japanese version of the National Adult Reading Test (33) measures the estimated intelligence quotient (IQ).

### 2.3. Iowa gambling task

We used the Japanese version of the IGT in the present study. Its only difference from the original IGT, which is presented by Bechara (11), is that the play money is in Japanese yen instead of US dollars. The participants chose one card at a time from four decks of cards labeled A, B, C, and D. They were told that they had a loan of JPY 200,000 (fictitious money) available at the start of the task, and they selected cards from any deck at their own pace. Decks A and B incur net losses over time. Although they provide larger immediate rewards (+10,000 yen for every card), they also impose larger penalties (a penalty of −125,000 yen after picking 10 cards). Deck A has five losses per 10 cards and Deck B has one loss per 10 cards. In contrast, decks C and D have total rewards (+5000 yen on every card) that are greater than the penalties (total penalty of −25,000 yen after picking 10 cards; deck C has five losses per 10 cards and deck D has one loss per 10 cards). The participants were not informed about the risks of each deck or the number of selections allowed. One hundred cards were selected to complete the task, which were subsequently divided into five blocks of 20 card selections.

Decision-making was evaluated using the total net score and the net score of each block. The net score was computed by subtracting the number of cards in each block of 20 card selections from the total number of cards. To investigate decision-making changes over time, we calculated the net score of each block and the total blocks.

### 2.4. Prospect valence learning model

The PVL model is comprised of two models based on the learning model. One is the PVL model with a delta-learning rule (PVL-Delta) which uses the Rescorla-Wagner rule (34), and the other is the PVL model with a decay-reinforcement learning rule (PVL-DecayRI). Several studies reported that the PVL-DecayRI had better *post-hoc* model fits than the delta rule on the IGT (21, 35, 36); therefore, this study adopted the PVL-DecayRI.

The explanation of the PVL-DecayRI, which is described below, has been cited in previous studies (21–25, 37, 38).


$$\begin{array}{l} u\;(t)=\{\begin{array}{c} \;x\;{\left(t\right)}^{\alpha }\;if\;x\;(t)\geq 0\;\\ -\text{λ}|x\left(t\right){|}^{\alpha }\;if\;x\;(t)<0\; \end{array} \end{array}$$

The above calculation shows how a participant's subjective expectancy valence [u (*t*)] is developed. Here, *x* (*t*) is the net gain, which is defined as *win* (*t*) – | *lose* (*t*)| in trial *t*, and α determines the feedback sensitivity. It has a value of zero to one and shows the relationship between the actual net gain and expectancy valence. As the value approaches zero, the individual's expectancy valence is not influenced by the actual number of gains or losses. If the value goes to one, the expectancy valence is sensitively influenced by the alternation of the actual amount of gains/losses. In this equation, λ is a loss aversion parameter, which ranges from zero to five and shows a tendency to respond sensitively to losses relative to gains. If λ = 0, the participant does not judge it as a loss at all. If λ = 1, the participant judges the losses and gains are equal, whereas if λ > 1, the participant tends to pay more attention to the losses than gains in the formation.

According to the PVL model, expectancy valence is affected by learning. This implies that the experience of gain or loss in the early trials influenced the expectancy valence of the participants. The recency parameter, which is the learning parameter in PVL-DecayRI, is calculated using the following equation:


$$\begin{array}{l} E_{j}\;\left(t\right)=A\cdot E_{j}\;\left(t-1\right)+I_{\text{j}}\;\left(t\right)\cdot u\;\left(t\right) \end{array}$$

In the above calculation, *E*_*j*_ (*t*) shows the expectancy for deck *j* on trial *t*. *A* is the recency parameter, which determines the earlier expectancy valence of a selected deck *j* on trial *t*. *I*_*j*_ (*t*) is a dummy variable. It is coded 1 if *j* card is selected in *t*th trial. If *j* card is not selected, *I*_*j*_ (*t*) is coded as zero. The recency parameter (0 < *A* < 1) indicates the extent to which the expected values of all decks are discounted in each trial. A low value of *A* means rapid forgetting and a strong recency effect, whereas a high A value indicates good learning/less memory decay.

The response consistency parameter is calculated using the following equation:


$$\begin{array}{l} \begin{array}{l} Pr[D(t+1)=j]=\frac{e^{\theta (t)\cdot E_{j}(t)}}{\sum_{k=1}^{4}e^{\theta (t)\cdot E_{k}(t)}} \end{array} \end{array}$$

*D* (*t* +1) is defined as the deck selected in the next trial, *t* + 1. The probability that deck *j* is selected for the next trial is given by *Pr* [*D* (*t*+1) = *j*]. Parameter θ (*t*) shows the degree of selection of the decks, which is formed by the expectancy valence. The PVL model adapts a trial-dependent choice rule which is showed θ (*t*) = 3^*c*^-1. The response consistency parameter *c* ranges from zero to five. If *c* approaches zero, the participant makes a random selection, whereas if c approaches five, the participant makes a consistent deck selection based on expectancy.

### 2.5. Statistical analysis

Tests were conducted using SPSS version 28.0.1.0. Demographic and clinical data were analyzed using chi-square test, Student's *t*-test, and Welch's *t-*test. To examine IGT performance, a two-way repeated measures ANOVA was performed as the between-subject factor and block as the within-subject factor. The Bonferroni correction was used for *post-hoc* analyses. Spearman's correlation test was used to examine the correlation between the total net score for OCD and the severity of OCD symptoms.

To estimate PVL parameters, a Markov Chain Monte Carlo sampling scheme was used in OpenBUGS, and BRugs was used (39). The PVL parameters were analyzed using a Mann-Whitney *U* test. Correlations between PVL parameters and the severity of OCD symptoms were analyzed using the Spearman correlation coefficient test.

## 3. Results

### 3.1. Demographic characteristics

There were no significant differences in sex [χ*2* (1) = 0.05; *p* = 0.826], age [*t* (92) = 0.18; *p* = 0.857], and estimated verbal IQ [*t* (92) = −1.48; *p* = 0.143] between the patients with OCD and HCs. However, HAM-D [*t* (66.74) = 5.15; *p* = 0.000] and HAM-A [*t* (52.51); *p* = 0.000] significantly differed. The mean Y-BOCS score in the OCD group was 23.81 (standard deviation, 5.97). Table 1 shows the mean scores of the demographic characteristics and clinical symptoms.

**Table 1:** Demographic and clinical characteristics [mean (standard deviation)].

	**OCD *n =* 47**	**HC *n =* 47**	**Statistics**			
			*χ**2***	***t***	***df***	***p-*** **value**
Sex, male/female	16/31	15/32	0.05		1	0.826
Age, years	36.19 (10.34)	35.76 (12.15)		0.18	92	0.857
IQ^a^	105.26 (7.52)	107.47 (6.83)		−1.48	92	0.143
HAM-D	5.09 (4.85)	0.98 (2.37)		5.16	66.74	*p* < 0.0001^***^
HAM-A	6.32 (7.46)	0.85 (1.43)		4.80	52.51	*p* < 0.0001^***^
Y-BOCS total	23.81 (5.97)	0.04 (0.20)		23.81	46.11	*p* < 0.0001^***^
Y-BOCS obsession	11.85 (3.49)	0.04 (0.20)		22.88	46.31	*p* < 0.0001^***^
Y-BOCS compulsion	11.96 (3.20)	0.00 (0.00)		25.38	46	*p* < 0.0001^***^
Duration of illness, month	126.21 (117.36)	NA				

HAM-A, Hamilton Anxiety Scale; HAM-D, Hamilton Depression Scale; IQ, intelligence quotient; JART, Japanese version of National Adult Reading Test; Y-BOCS, Yale-Brown Obsessive Compulsive Scale. ^a^Estimated IQ was measured with the JART. ^***^p < 0.001.

### 3.2. IGT performance

**Figure 1:**
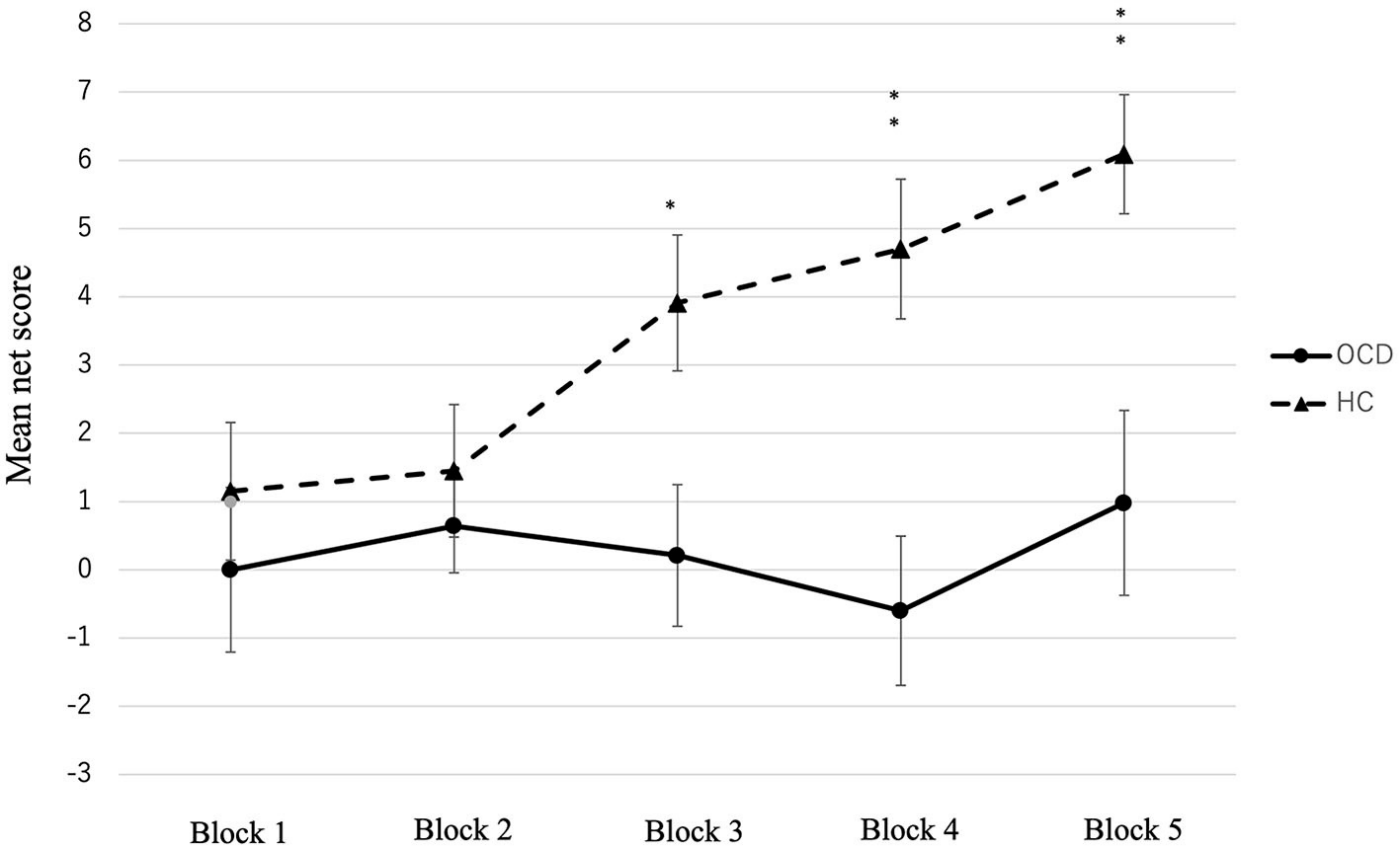
Mean net score of the five blocks in the OCD and HC. Significant differences were observed between the two groups in blocks 3, 4, and 5. *p < 0.05, **p < 0.001, ***p < 0.0001.

The total net score of the OCD group was significantly lower than that of the HC group [*t* (92) = −3.54, *p* < 0.001, *r* = 0.120] (Table 2). We conducted a two-factor repeated measures ANOVA on the conditions, and the results showed a significant difference in the group factor [*F*_(1, 92)_ = 12.54, *p* = 0.001, *partial* η^2^ = 0.120], block factor [*F*_(4, 368)_ = 3.11, *p* = 0.015, *partial* η^2^ = 0.033], and interaction effects (*F*_(4, 368)_ = 2.73, *p* = 0.029, *partial* η^2^ = 0.032]. Figure 1 shows the changes in net scores in the OCD and HC groups. In the main effect of the block factor, the net score of block 5 was significantly higher than that of block 1 (*p* = 0.019) using Bonferroni corrections. In the interaction, the simple main effects of the group factor showed significant differences: the net score of block 3 (OCD < HC, *p* = 0.012, *partial* η^2^ = 0.067), block 4 (OCD < HC, *p* < 0.001, *partial* η^2^ = 0.120), and block 5 (OCD < HC, *p* = 0.002, *partial* η^2^ = 0.098). In the HC, the simple main effect between blocks was significantly different [*F*_(4, 89)_ = 4.95, *p* = 0.001, *partial* η^2^ = 0.182]. The *post-hoc* comparison using Bonferroni corrections in the HC group showed that the net score of block 5 was significantly higher than that of blocks 1 (*p* = 0.03) and 2 (*p* = 0.003). Meanwhile, in OCD, there were no significant differences in the simple main effect between each block [*F*_(4, 89)_ = 0.54, *p* = 0.709, *partial* η^2^ = 0.0249].

**Table 2:** IGT performance of OCD and HC [mean (standard deviation)].

	**OCD (*n =* 47)**	**HC (*n =* 47)**	***F***	***t***	***p*-value**	**Effect size**
**IGT**						
Block 1	0.00 (8.18)	1.15 (6.83)	0.534		0.467	0.006
Block 2	0.64 (4.63)	1.45 (6.58)	0.464		0.498	0.005
Block 3	0.21 (7.04)	3.91 (6.75)	6.624		0.012^*^	0.067
Block 4	−0.60 (7.41)	4.70 (6.94)	12.514		*p* < 0.001^***^	0.120
Block 5	0.98 (9.19)	6.09 (5.92)	10.041		0.002^**^	0.098
Total	1.23 (22.50)	17.28 (20.98)		−3.54	*p* < 0.001^***^	0.120

^*^p < 0.05, ^**^p < 0.01, ^***^p < 0.001.

There was no significant correlation between the total net score and the obsessive Y-BOCS score (*r* = 0.160, *p* = 0.273), compulsive Y-BOCS score (*r* = 0.226, *p* = 0.119), or total Y-BOCS score (*r* = 0.222, *p* = 0.126).

### 3.3. PVL model parameters

**Figure 2:**
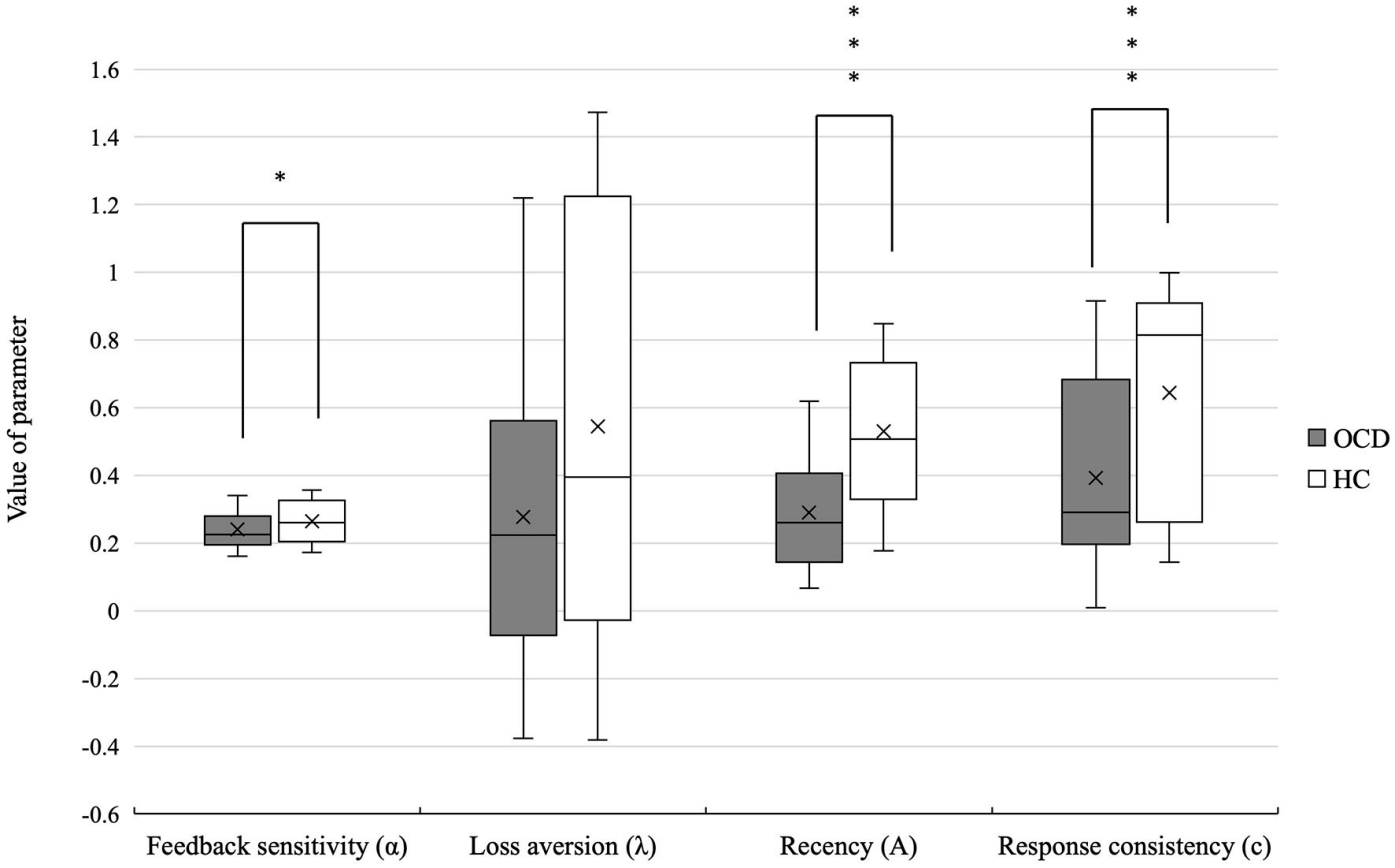
Differences in the PVL-DecayRI model parameter of OCD and HCs. Significant differences were observed between the two groups in feedback sensitivity, recency, and response consistency. *p < 0.05, ***p < 0.001.

Patients with OCD showed lower feedback sensitivity (*U* = 1373.5, *p* = 0.042, *r* = 0.21), recency (*U* = 17770.0, *p* = 0.000, *r* = 0.52), and response consistency (*U* = 1625.0, *p* = 0.000, *r* = 0.41) than the HCs. There was no significant difference in loss aversion (*U* = 1361.5) parameter between the two groups. The mean PVL parameter values in patients with OCD and HCs are shown in Table 3 and Figure 2.

**Table 3:** Mean value of PVL-DecayRI model parameters of OCD and HCs.

**PVL parameters**	**OCD (*n =* 47)**	**HCs (*n =* 47)**	***U*-value**	***Z* score**	***p*-value**	***r***
Feedback sensitivity (α)	0.240 (0.054)	0.265 (0.061)	1373.5	2.034	0.042^*^	0.21
Loss aversion (λ)	0.278 (0.389)	0.545 (0.597)	1361.5	1.943	0.052	
Recency (*A*)	0.290 (0.177)	0.530 (0.213)	1770.0	5.033	*p* < 0.001^***^	0.52
Response consistency (*c*)	0.393 (0.280)	0.645 (0.305)	1625.0	3.936	*p* < 0.001^***^	0.41

PVL-DecayRI, Prospect Valence Learning decay reinforcement learning rule; ( ), standard deviation; ^*^p < 0.05, ^***^p < 0.001.

**Figure 3:**
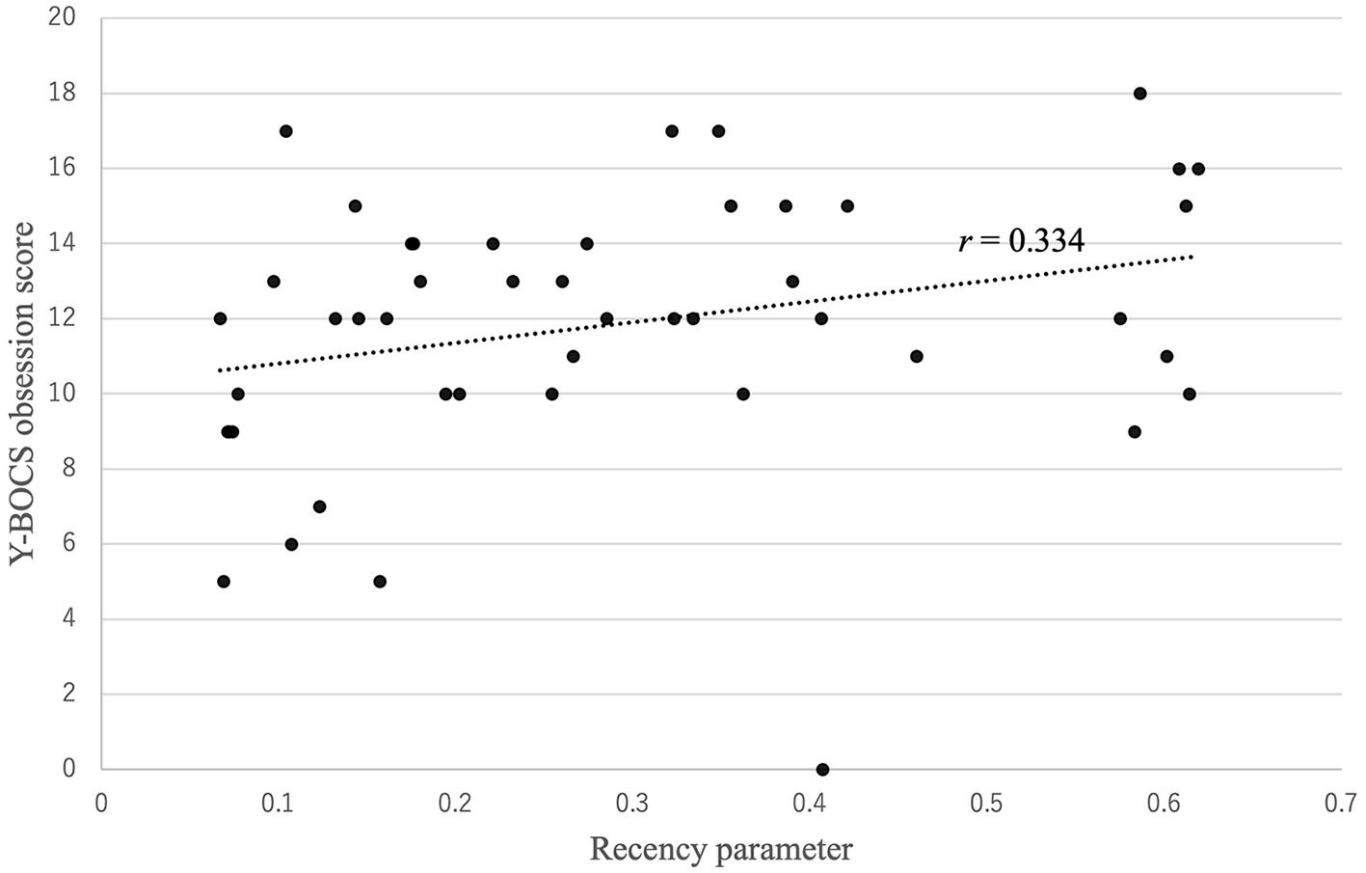
Correlation between the recency parameter and Y-BOCS obsession score. There was a positive correlation between the recency parameter and Y-BOCS obsession score: *r* (47) = 0.334, *p* = 0.022.

**Figure 4:**
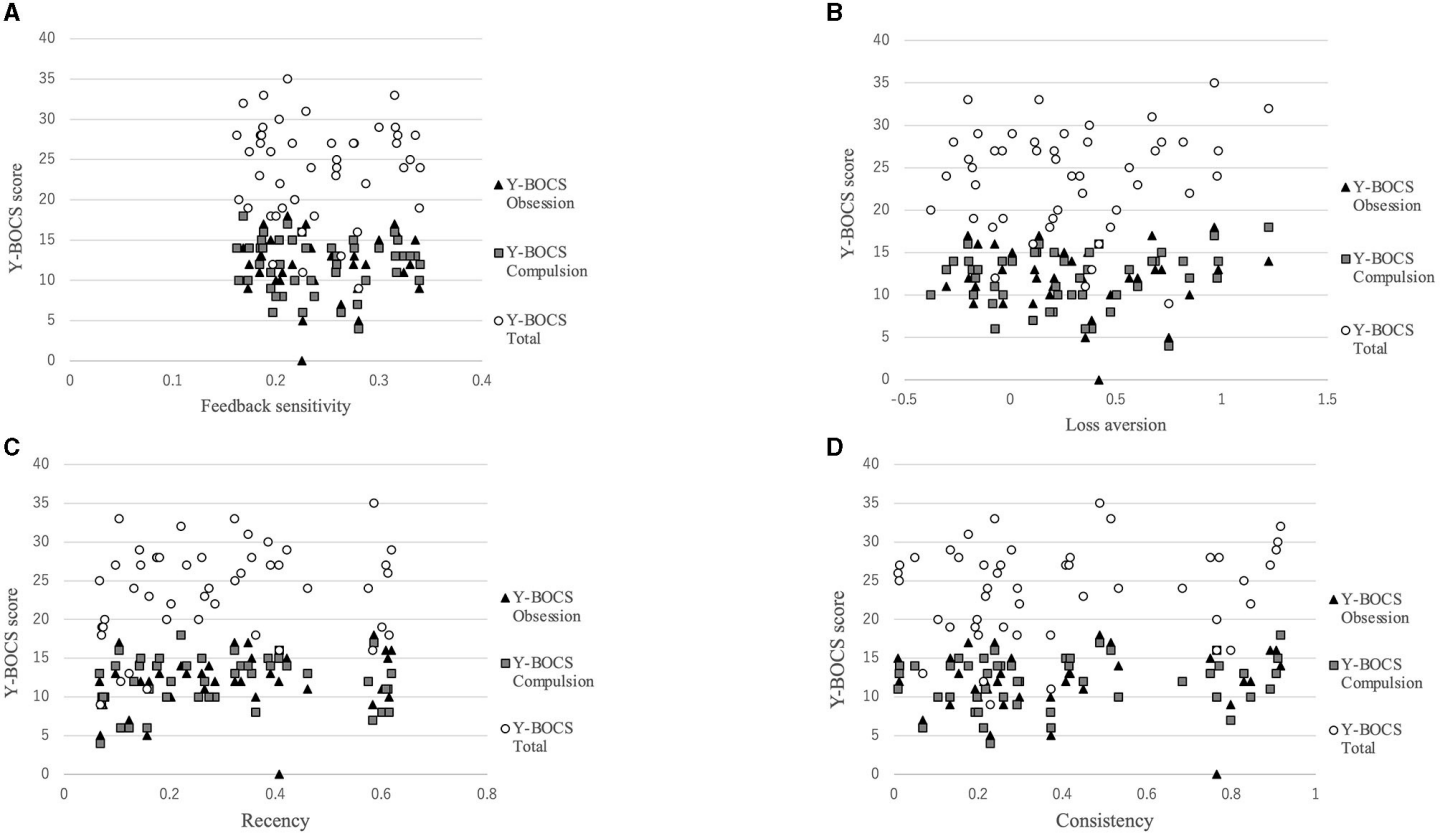
Correlations between symptom severity and each parameter for the PVL-DecayRI. Except for that between the Y-BOCS obsession score and recency parameter, no correlation was observed between the Y-BOCS scores and the other parameters for the PVL-DecayRL. **(A)** Feedback sensitivity parameter; **(B)** loss aversion parameter; **(C)** recency parameter; and **(D)** consistency parameter.

Figure 3 shows that there was a positive correlation between the recency parameter and Y-BOCS obsession score (*r* = 0.334, *p* = 0.022). No correlations were observed between the other PVL parameters and OCD symptom severity (see Figure 4). The correlation coefficients are presented in Table 4.

**Table 4:** Correlations between symptom severity and each parameter on PVL-DecayRI.

	**Feedback sensitivity**	**Loss aversion**	**Recency**	**Consistency**
**Y-BOCS**				
**Obsession score**				
*r*	0.022	0.065	0.334	0.173
*p*	0.881	0.664	0.022^*^	0.246
**Compulsion score**				
*r*	−0.114	0.116	−0.154	0.182
*p*	0.446	0.439	0.301	0.22
**Total score**				
*r*	−0.045	0.067	−0.221	0.169
*p*	0.762	0.656	0.136	0.257

PVL-DecayRI, Prospect Valence Learning decay reinforcement learning rule; ( ), standard deviation; ^*^p < 0.005.

## 4. Discussion

We found that patients with OCD had a deficit in decision-making and significantly lower recency and response consistency parameters than those in the HC group. However, the loss aversion parameter was not significantly different between patients with OCD and HC. In line with a previous meta-analysis (15), patients with OCD showed significantly lower total net scores than HC, no increase in net scores as the trials progressed, and no correlation between the total net score and symptom severity. Value-based decision-making, including the IGT, incorporates emotion-based learning and interoception (40, 41). IGT performance encompasses both emotional and cognitive processes, which are facilitated by reward- and punishment-related motivation (42). Individuals with OCD encounter challenges in emotion regulation (43) and deficits in interoception (44, 45) and exhibit impaired reward generalization (46). Therefore, the OCD group exhibited poorer IGT performance compared with the HC group.

The lower recency and response consistency parameters in the OCD group indicate that patients with OCD had difficulty updating the current outcome and selected the cards randomly. Therefore, patients with OCD either have deficits in learning about eventuality-controlling options (25, 47) or experience a rapid decline in previously learned information (25, 48). In addition, patients with OCD may impulsively choose cards throughout the five blocks (25, 48).

These results may be due to structural and functional brain abnormalities associated with OCD. Previous studies have revealed that the brain regions related to IGT performance in IGT are the orbitofrontal cortex (OFC) (49–51), dorsolateral prefrontal cortex (51, 52), ventromedial prefrontal cortex (51–54), anterior cingulate cortex (52), and parietal cortex (52). Additionally, striatal circuits are associated with learning and decision-making (55). Patients with OCD exhibit structural and functional abnormalities (56–61) in these brain regions. Moreover, the working memory (WM) deficits in patients (62–64) might be associated with the results of this study. The WM is the cognitive system used to hold some amount of information as the focus of attention (65, 66) and is associated with IGT performance (66–68). The prefrontal and parietal cortices are involved in WM (69–71), and patients with OCD exhibit dysfunction and structural abnormalities in these regions (56, 61, 72). Thus, these abnormalities in the brain regions may involve lower values of recency and consistency parameters.

The reduced feedback sensitivity observed in the OCD group suggests that the subjective evaluation of deck selection outcomes among OCD patients was not significantly influenced by the actual amount of gains or losses. This could potentially indicate a deficit in the reward processing system, linked to the disruption of frontostriatal circuits in OCD (73, 74). However, the effect size of this difference was very small. Therefore, the results should be interpreted with caution.

Contrary to our initial hypothesis suggesting a higher loss aversion parameter among OCD patients than HCs, the loss aversion parameter in the OCD group was not significantly different from that of HCs. This finding indicates that the lower IGT performance in individuals with OCD does not involve loss aversion. Interestingly, certain addictive disorders associated with compulsive behaviors (75), such as Internet gambling disorder, pathological gambling disorder, and alcohol disorder, demonstrated lower performance on value-based decision-making and lower loss aversion parameters of the prospect theory compared with HCs (76, 77). Although no direct correlation was established between the loss aversion parameter and severity of compulsivity in this study, compulsivity could influence our results. Further research is required to validate our results and hypotheses.

We found no correlation between the total net score and symptom severity in patients with OCD. These results are supported by a previous meta-analysis (4), which proposed that the deficit in decision-making might be an endophenotype of OCD. Interestingly, in this study, the value of the recency parameter in patients with OCD, which was lower than that in HC, was positively correlated with the Y-BOCS obsessive score. In other words, the more severe the obsession OCD patients have, the larger the influence on the expectancy of the most recent outcome. Thus, the lower recency parameter may be a state, but not a trait, of OCD. Conversely, the value of the consistency parameter, which was lower than that of HCs, did not correlate with symptom severity. As mentioned above, the lower consistency parameter in patients with OCD indicates that OCD patients might make impulsive choices under ambiguous decision-making conditions (25, 48). Some studies have found that impulsivity and compulsivity may be involved in common psychological and neurobiological mechanisms (78–81) and have proposed that impulsivity might be a feature of OCD (82, 83). Therefore, the lower consistency parameter in patients with OCD may reflect their trait nature.

This study has some limitations. First, we did not examine variables such as reward sensitivity, approach-avoidance motivational tendencies, or interoception, all of which affect IGT performance. Therefore, our findings could be influenced by these unexplored factors. Second, we did not identify the best-fitting model by comparing it with other cognitive models, such as the Value-Plus Perseverance model (84) and outcome representation learning model (85). However, this study aimed to compare the values of PVL-DecayRI parameters between patients with OCD and HCs. Future studies are needed to develop the best-fitting model for IGT in OCD. Third, we did not employ neuroimaging techniques; nevertheless, we mentioned some brain abnormalities in patients with OCD in the Discussion section. Fourth, we did not examine aspects of OCD heterogeneity such as age at onset (86–88), duration of illness (89), and OCD dimensional symptoms (90, 91). Fifth, we did not investigate other neuropsychological functions associated with IGT, such as working memory, attention, and set-shifting. Despite these limitations, we found new information regarding decision-making in OCD. As mentioned above, some studies have proposed that decision-making may be one of the candidates for the endophenotype of OCD (4). In future, we hope to address the limitations of this study and conduct a study to investigate the parameters within a computational model for individuals who have relatives with OCD to determine whether the same parameters, observed in individuals with OCD, are also involved in their reduced IGT performance (4).

## 5. Conclusion

Patients with OCD had significantly lower total net scores than HC, and there was no increase in net scores as the trials progressed on the IGT. Application of the PVL-DecayRI model showed that patients with OCD had lower recency and response consistency parameters than the HC group. These findings indicate that a deficit in decision-making in OCD may occur because the most recent outcome has a small effect on expectancy, thereby contributing to a computational decision-making model for patients with OCD.

## Data availability statement

The original contributions presented in the study are included in the article/supplementary material, further inquiries can be directed to the corresponding author.

## Ethics statement

The studies involving humans were approved by Kyushu University Ethics Committee. The studies were conducted in accordance with the local legislation and institutional requirements. The participants provided their written informed consent to participate in this study.

## Author contributions

KM designed the study, collected the data, and wrote the initial draft of the manuscript. HT collected the data, designed the study, and critically reviewed the manuscript. AO, MK, and KS contributed to the data analysis and interpretation. SH, KK, and AM contributed to data collection. TN critically reviewed the manuscript. All authors approved the final version of the manuscript.
